# Evaluating building stones: Physical-mechanical changes from high-temperature fire and water cooling

**DOI:** 10.1016/j.heliyon.2024.e36108

**Published:** 2024-08-10

**Authors:** Vera Pires, Fabio Sitzia, Carla Lisci, Licinio Cordeiro

**Affiliations:** a HERCULES Laboratory and IN2PAST, Associate Laboratory for Research and Innovation in Heritage, Arts, Sustainability and Territory. Institute for Advanced Studies and Research. University of Évora. Largo Marquês de Marialva 8, 7000-809, Évora, Portugal; b Geosciences Department, School of Sciences and Technology, University of Évora, Rua Romão Ramalho 59, 7000-671, Évora, Portugal; c LEM Laboratório de Ensaios Mecânicos da Universidade de Évora, R. Romão Ramalho 59, 7000-671, Évora, Portugal; d AIRELIMESTONES, Rua dos Arneiros, Ataíja de Cima, 2460-713, Alcobaça, Portugal

**Keywords:** High temperature, Fire, Physical-mechanical properties, Durability, Limestone

## Abstract

Heritage sites built with natural stone are at risk from fires, which can alter stone properties and compromise its structural integrity. Over 60 studies in the past three decades have examined fire impact on natural stone, providing insights for their prevention and restoration. The primary objectives are to develop effective strategies to mitigate fire risks, protect heritage structures, and ensure the preservation of our cultural legacy. Two noteworthy Portuguese limestones used as heritage building materials: Lioz (LL) and grey Ançã stone (GAS), were studied regarding the effect of high-temperature exposure for simulating fire at 200 °C, 400 °C and 600 °C, followed by cooling in water to reproduce fire extinguish in natural stone buildings. The findings provided insights into how the different temperatures impact the stone morphological, physical and mechanical properties. Color measurements (CIE L*a*b*) showed a color difference from 3 to 32 %; SEM-EDS confirmed microstructure modifications after fire exposure with cracks formation and intragranular porosity development. Among the diverse physical and mechanical properties of the stones, uniaxial compressive strength decreased from 1 to 33 %, Leeb D hardness decreased up to 12.2 %, lowering in open porosity was detected in the range 70–289 % and ultrasound speed propagation were significantly affected after thermal cycle at 600 °C with a negative variation reaching 49 %. Results from TGA show a loss of mass due to retained water (∼40 °C) and loss of hydration water at ∼ 120 °C in both limestones. The total mass loss (∼42–∼44 %) is associated with the loss of H_2_O, CO_2_. In conclusion, stones with higher toughness and compression strength exhibited reduced damage at high temperatures due to their enhanced resistance to fracturing under stress. As limestone's mechanical strength decreases under high temperatures, it's advisable to increase its thickness to ensure sufficient support for loads and intended conditions of use. The deficiency of analysis on limestone's mechanical decay from fire reveals a significant knowledge gap regarding the complete extent of damage and deterioration in stone heritage structures.

## Highlights

• Microstructure analysis uncovers microcrack propagation and pore enlargement at high temperatures.
• Thermal shock reduces hardness and ultrasonic pulse velocity, impacting compression strength.
• Temperature effects on limestone properties are crucial for structural design and durability.

## 1 Introduction

Natural stone, admired for its enduring beauty and historical significance in cultural heritage, faces the pervasive threat of hazards, with fire emerging as a particularly damaging force. The intersection of these elements underscores the need for a comprehensive understanding of the vulnerabilities of natural stone building materials when exposed to fire.

Heritage sites, often constructed with meticulous craftsmanship using natural stone, are vulnerable to the destructive impact of fires. The intense heat generated during a fire can induce profound changes in the physical and mechanical properties of these stones, jeopardizing their structural integrity.

The study of fire as a damaging agent to natural stone in heritage structures is pivotal for preserving our cultural legacy. In the last three decades, more than 60 investigations about the effects of fire on stone materials contribute important insights into preventive measures and restoration strategies [[1], [2], [3], [4], [5], [6], [7], [8], [9]].

One key area of research has focused on the thermal behavior of different types of natural stone, such as limestone, sandstone, and granite. Studies have shown that fire exposure can cause considerable changes in the physical and chemical properties of these materials. For instance, when exposed to high temperatures, limestone tends to undergo calcination, leading to a loss of structural integrity and increased porosity. Sandstone, depending on its composition, can suffer from cracking and spalling due to differential thermal expansion of its mineral constituents. Granite, while generally more resistant, can also experience microcracking and discoloration when subjected to extreme heat [1,10,11].

In addition to laboratory studies, in-situ assessments of fire-damaged heritage sites have provided valuable insights. For example, post-fire evaluations of historical buildings have demonstrated that fire can exacerbate pre-existing structural weaknesses and accelerate weathering processes. These findings underscore the importance of integrating fire risk assessments into the conservation strategies for stone heritage sites [2,7,[12], [13], [14], [15], [16], [17]]. Furthermore, research has explored mitigation and preservation techniques to protect natural stone from fire damage. These include the application of fire-retardant coatings and the development of advanced monitoring systems to detect early signs of thermal distress. Such measures are crucial for enhancing the resilience of both existing stone structures and new constructions [18].

By assessing the hazards posed by fire and the subsequent damage inflicted on natural stone, researchers and conservation scientists can develop informed strategies to mitigate risks and safeguard the longevity of heritage structures [2,19].

The role of fire, accompanied by exposure to elevated temperatures, is unequivocally acknowledged as a catalyst for alterations in buildings [8,[20], [21], [22], [23], [24]]. Varied levels of fire risk are associated with routine activities, construction processes, and human-related factors such as the use of lit candles, batteries, fireplaces, or poorly maintained chimneys [18].

Previous research has conclusively demonstrated that the primary outcomes of stone degradation of international monuments triggered by fire are predominantly shaped by the lithological characteristics. Some example are: Notre-Dame of Paris [25], fire at Heidelberg Castle (Heidelberg, Germany) Church of our Lady (Dresden, Germany) [15], fire at Lisbon Cathedral [21]. The recent damage caused by fire at recent fire at Denmark's Boersen Building is currently under investigation. These aspects encompass the mineral composition, texture, and structural attributes of the stone. Interestingly, the extent of stone deterioration appears to exhibit a more substantial correlation with these inherent geological features rather than being significantly influenced by the specific conditions of the fire event. This insight underscores the fundamental role played by the intrinsic nature of the stone itself in determining the impact of fire-induced decay, providing information for assessing and mitigating potential risks to building materials during fire incidents [8,21,24,26].

While stone is commonly regarded as a highly resilient construction material due to its non-combustible nature, it is essential to recognize that fire can transfer heat to stone materials through thermal radiation, conduction, and convection. In the classification provided by Hajpál and Török [27], fires are categorized into two main types based on their development and temperatures: (i) small, restricted fires, which typically generate lower heat (temperatures usually below 800 °C); and (ii) large, widespread fires with higher temperatures, reaching maximum levels around 1200 °C. The influence of such fires on the properties of stone can exhibit substantial variations, even when dealing with identical types of stone material.

Moreover, previous research indicates that using water to extinguish fire or cool stones, particularly carbonate stones, can pose additional challenges [20]. Below 600 °C, the thermo-metamorphism of limestones primarily stems from the markedly anisotropic thermal expansion of their main mineral phase (Calcite, CaCO_3_). This anisotropy, with positive expansion along the crystallographic c-axis and negative expansion in perpendicular directions, induces intergranular microcracking [17,28]. At elevated temperatures (frequently 600–950 °C), the limestone undergoes to the thermo-metamorphism by forming calcium oxide (CaO, quicklime). When quicklime is in contact with water, it readily reacts by forming portlandite Ca(OH)_2_ by an exothermic reactions [13]. This information underscores the nuanced dynamics of how different fire scenarios, and the subsequent use of water can affect the properties of stone materials, emphasizing the need for a comprehensive understanding in fire safety assessments and mitigation strategies.

In essence, the exploration of the nexus between natural stone, selection and dimensioning, hazards, heritage, and fire unveils a multifaceted challenge that demands interdisciplinary research and a commitment to the preservation of our collective past. As we delve deeper into these dynamics, we equip ourselves with the knowledge necessary to protect and conserve the invaluable legacy embedded in the stones of our cultural heritage.

This study provides a novel and comprehensive exploration of the physical and mechanical properties of two prominent Portuguese limestones, Lioz (LL) and grey Ançã stone (GAS), after exposure to high temperatures (600 °C, 400 °C, and 200 °C) followed by water cooling. The two lithotypes are, from ancient times, used in vernacular architecture as other stone materials in Mediterranean area [[29], [30], [31]]. By simulating the effects of fire extinction, the research addresses a critical gap in understanding the behavior of natural stones under such conditions. Key innovative aspects include.

1. High-Temperature Impact Assessment: The study uniquely investigates the impact of high temperatures on the dimensioning and application of these stones, an area with limited existing research.
2. Comprehensive Mechanical Evaluation: Parameters such as uniaxial compression strength and Leeb D hardness are assessed, which are scarcely covered in current literature [32].
3. Multi-Faceted Analysis: The research combines visual inspections, porosity analysis, ultrasonic speed propagation, CIE L*a*b* color measurements, thermo-gravimetric analysis, and microstructural modifications using SEM-EDS, both before and after heat exposure.
4. Focus on Fire Resilience: By understanding the impact of fire on natural stones, the study contributes to more informed material selection and design, enhancing safety, durability, and resilience against fire hazards in buildings.

Understanding the impact of fire on natural stone is paramount when dimensioning a building element, as it ensures informed decision-making in material selection and design, contributing to enhanced safety, durability, and resilience against potential fire-related hazards. This research offers valuable insights into the effects of high temperatures on structural and aesthetic properties of limestones, aiding in the development of safer and more resilient building practices.

## 2 Lioz limestone – a building and heritage stone

**Fig. 1:**
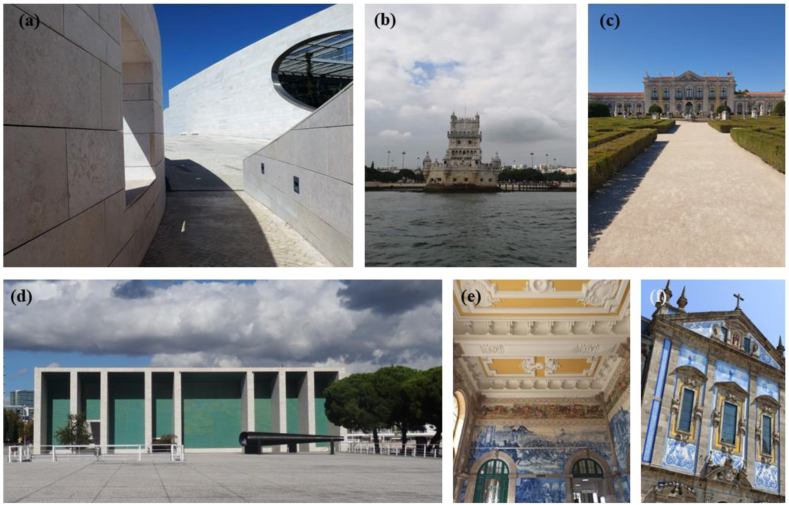
Lioz limestone (LL) and Ançã stone (AS) used in iconic building such as: **(a)** the Champalimaud research center for the unknown in Lisbon - Building exterior cladding with honed Lioz slabs, **(b)** Belém Tower built entirely by Lioz limestone (Belém, Portugal), **(c)** Queluz national palace - Ançã stone balustrades (Queluz – Portugal), **(d)** Portugal pavilion – Building exterior cladding with honed slabs of Lioz (Lisbon, Portugal)**, (e)** and **(f)** São Bento train station - Interior cladding with ançã stone honed finishing (Porto, Portugal). Photos by Vera Pires and Carla Lisci.

LL is renowned for its exceptional qualities, has been a favored material in the construction and preservation of heritage structures for centuries. Quarried in Portugal (*Pero Pinheiro* region), Lioz limestone boasts a distinctive aesthetic appeal characterized by its warm beige to golden hues, intricate bioclast patterns, and a unique texture that exudes timeless elegance. One of the distinctive features of LL lies in its versatility, making it an ideal choice for a wide range of architectural elements in heritage construction. From ornate facades and complex sculptures to grandiose columns and carvings, LL has been utilized to shape and embellish some of the world's most iconic landmarks, some of them depicted in Fig. 1, such as: the Jerónimo's monastery, the Belém tower, the Portugal pavilion, the Champalimaud research center for the unknown in Lisbon (Portugal), Mafra Architectonic complex (library, convent, and church) also in Portugal, São Pedro dos Clérigos church and Ordem Terceira de São Francisco and São Francisco church and convent all located in Bahia, Brazil [33,34]; some of these are depicted in Fig. 1. Its durability and resistance to weathering contribute significantly to its popularity as a heritage construction material. LL weathers gracefully over time, developing a natural patina that enhances its aesthetic appeal while maintaining structural integrity. This characteristic makes it well-suited for exterior applications, ensuring longevity and sustainability in the face of environmental exposure [[33], [34], [35], [36], [37]]. Furthermore, the workability of LL allows for intricate detailing and precision carving, making it a preferred choice for artisans and craftsmen involved in heritage restoration projects. Its adaptability to various carving techniques enables the creation of intricate reliefs and decorative elements, adding a touch of sophistication to architectural masterpieces [36]. LL stands as a testament to the enduring legacy of heritage construction materials. Its innate beauty, durability, and historical significance make it a timeless choice for those seeking to safeguard and perpetuate the cultural and architectural heritage embedded in our built environment. As a result of the attributes, LL has been officially recognized as a Heritage Stone by the International Union of Geological Sciences (IUGS) (https://iugs-geoheritage.org/geoheritage_stones/lioz-limestone/). The weathering of LL has been previously studied and the decay is commonly determined by its inherent attributes: its low porosity and fine-grained texture on one side, and the eventual occurrence, percentage, and spatial distribution of clay minerals, iron oxides, and stylolites on the other [36,[38], [39], [40], [41]]. The most refined variations exhibit commendable performance in urban settings and demonstrate resilience against both salt decay and thermal influences. While the natural dissolution of calcite, typically a minor decay mechanism, ultimately assumes a pivotal role, underscoring the robustness and longevity inherent in these varieties [34,42]. The particularity and novelty of this work are given by the study of two stones of global importance both nominated as recognized as a World Heritage Stone by the IUGS sub-commission Heritage Stones, an organization under UNESCO.

The designation attests to the geological, paleontological, cultural, sociological, and historical significance of both the limestone, as well as its artistic value. In Portugal, Ançã and Lioz limestones joins the ranks of Estremoz marble and Arrábida breccia, which have already been designated as World Heritage Stones.

## 3 Ançã stone – a heritage stone

The rock usually referred to as AS is a cream to greyish and homogeneous limestone dating back to the Bajocian-Bathonian period (lower Jurassic), forming part of the Ançã Formation. Recognized for its exceptional workability, including suitable resistance for masonry units and slabs for wall cladding and flooring applications, high porosity, and water absorption, AS has played a significant role in decorating architectural heritage such as churches, palaces, and monumental constructions. Having been extensively used in sculpture, AS experienced a peak in popularity during the 16th century, particularly with the renowned sculptors João de Ruão and Nicolau Chanterene. From 1867 to 1927, quarrying activities for lime production, masonry, and sculptures led to industrial development in the region, contributing to the growth and prosperity of Cantanhede municipality [43].

The use of AS is prominent in several buildings in Coimbra, including the University's Porta Férrea, Porta Especiosa at Sé Velha and the Church of Santa Cruz. Its application extends beyond Coimbra to various national and international monuments, reflecting its enduring significance: Queluz national palace (Queluz, Portugal) (Fig. 1), São Bento Station (Porto, Portugal), Capela da Santissima Trindade in Quinta da Regaleira located in Sintra (Portugal) are just some examples.

However, the stone susceptibility to decay, particularly due to porosity and water absorption, has prompted extensive research over the years. Several research works have delved into various aspects of the AS, contributing to a comprehensive understanding of its characteristics and behavior when exposed to atmospheric agents [21,[43], [44], [45], [46], [47], [48]].

Recognizing the importance of preserving this heritage, the municipality of *Cantanhede* established a stone Museum in 2001, showcasing the diverse perspectives of AS. The culmination of these efforts, including research, documentation, and the creation of the Stone Museum.

## 4 Experimental studies

### 4.1 Materials

All specimens of Lioz limestone (LL) and grey Ançã stone (GAS) underwent cutting using a circular diamond saw and were finished by honing.

**Fig. 2:**
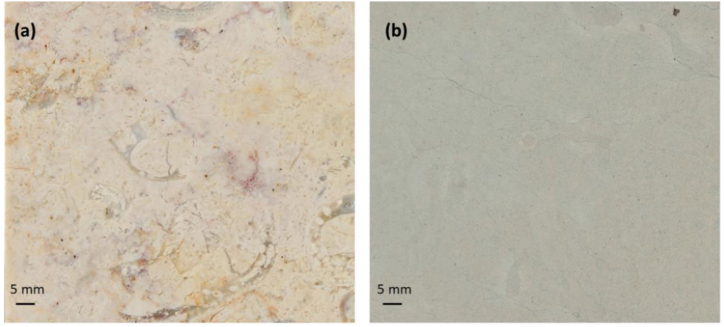
Macroscopic characteristics of the studied limestones: (a) Lioz; (b) Ançã stone.

LL limestone is a compacted, light cream - yellowish microcristalline bioclastic and heterogeneus limestone, distinguished by stylolithic joints exhibiting variable opening and spacing depending on the *facies* (Fig. 2a) [34]. LL is extracted in the following location: 38°51′34.3″N 9°18′56.6″W. This quarry is in Pero Pinheiro, Municipality of Sintra.

GAS is an homogeneous greyish micritic, relative soft, porous and marly limestone sharing common geological characteristics with various lithotypes of Ançã Stone globally employed in architecture [45,48,49] (Fig. 2b). GAS, is extracted from the following location: 40°17′10.7″N 8°35′41.4″W. Quarry is situated in Outil, Municipality of Cantanhede, Portugal. This quarry provides a stone material of light beige color which is representative of the Ançã stone recognized by UNESCO as a Global Heritage Stone Resource. Additionally, there exists a lateral variation of this primary material which belongs to the same stratigraphic unit—a greyish, more resilient stone with lower porosity (GAS). The application to UNESCO includes this information about possible variations from cream to grey within the same unit. Based on these observations and the information gathered on-site, the authors did not consider the analyzed material to be geologically distinct from Ançã stone; rather, it is viewed as different facies of the same unit. Studied limestones reference physical-mechanical properties are depicted in Table 1. More results of physical-mechanical properties can be found on the Portuguese ornamental stone catalogue [50].

**Table 1:** Reference physical-mechanical properties of LL and GAS (data supplied by stone manufacturers).

Properties, normative/Stone type	LL	GAS
Flexural Strengh [MPa] | EN 12372	11.5 ± 2	15 ± 1.6
Apparent Density [kg/m^3^] | EN 1936	2702 ± 11	2370 ± 10
Open Porosity [%] | EN 1936	0.5 ± 0,2	10.2 ± 0.7
Water Absorption at Atmospheric Pressure [%] | EN 13755	0.2 ± 0.1	4.9 ± 0.1
Water Absorption by Capillarity [g/m^2^.s^0.5^] | EN 1925	0.56 ± 0.19	13.61 ± 0.12
Wear abrasion [mm] | EN 14157	20.5 ± 2.0	31.5 ± 2.3

To assess and characterize the impact of high temperatures followed by shock cooling with water on the stones' performance, a series of specimens were prepared with the following dimensions: 40 cubic specimens of each lithotype measuring 50 × 50 × 50 mm. These specimens were extracted from blocks, subjected to sawing, honing, washing, dried at 70 °C for 72 h in a ventilated oven, and subsequently stored in controlled conditions (20 °C and 50 % relative humidity).

### 4.2 Methods

Specimens were subjected to heat treatment in a Nabertherm muffle furnace with a maximum temperature capability of 1100 °C, operating in an oxidizing environment. Heating was carried out at 200, 400, and 600 °C for a duration of 24 h to simulate the thermal impact that occurs during a fire, ensuring uniform attainment of the maximum temperature across all samples thickness. The heating rate 3.3 °C/min during the heating phase of 1 h afterwards temperature was kept constant for 24 h. These selected temperatures were determined based on our understanding of the thermal metamorphism temperatures of the chosen stone (approximately 700 °C), which significantly affect the structural cohesion of the material. Additionally, we considered relevant reference works [20,26]. The warming-up phase spanned 60 min and following the 24-h exposure at each temperature, immediate cooling to room temperature was induced through tap water, mimicking the thermal shock experienced during the extinguishing stage of a fire.

Petrographic analyses were made with the help of the Hirox-01 digital microscope on of 30 μm thickness thin sections.

**Fig. 3:**
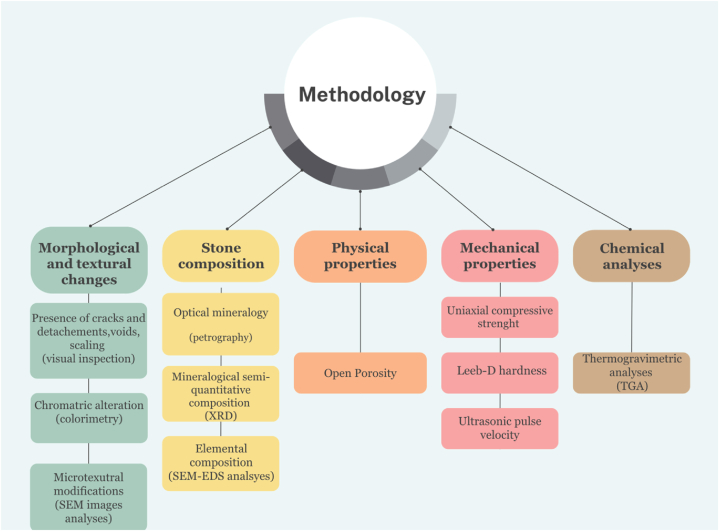
Simplified flow chart of methodology.

A simplified flow chart of experimental methodology is portrayed in Fig. 3.

#### 4.2.1 Morphological and textural changes

1) Visual inspection allowed to evaluate the possible formation of cracks, occurrence of detachments and scaling due to exposure to high temperatures and rapid cooling in fresh water.
2) Color information was digitally gathered from the stones using a HIROX digital microscope, and the data are referred to the CIE L*a*b* color space. The L* values denote luminosity, whereas a* and b* represent chromaticity coordinates: +a* corresponds to red, -a* to green, +b* to yellow, and –b* to blue. Color variations can be assessed using the following equation, Eq. (1) which is imposed by the standard EN 15886:2010. Conservation of cultural property - Test methods - Color measurement of surfaces (EN 15886:2010):

1 ΔL* = L_1_* - L_0_*; Δa* = a_1_* - a_0_*; Δb* = b_1_* - b_0_*

where: *L*_1_*, *a*_1_*, *b*_1_* are the final values, and *L*_0_*, *a*_0_*, *b*_0_* are the reference values.

The total color difference was determined as follows, Eq. (2):

ΔE* = (ΔL*^2^+Δa*^2^+Δb*^2^)^1/2^

3) SEM-EDS analyses were performed by a scanning electron microscope HITACHI S3700N (Hitachi High-Technologies Corporation, Tokyo, Japan). The observations were carried out on fragments of non-exposed and, after exposure to 200, 400 and 600 °C. This technique allowed to analyze and compare the combined effects of elevated temperature and subsequent water-cooling on the stones microstructure and obtain the elemental composition.

#### 4.2.2 Stone composition

1) Petrographic methods involve the microscopic examination of thin sections of stone samples by optical microscopy (OM) to analyze their mineral composition, texture, and microstructure. Observations were yielded by an Hirox HRX-01 digital microscope on thin sections of 30 μm thickness.
2) The mineralogical characterization of pre-tested stones was conducted using the X-ray diffraction technique (XRD analyses). A Bruker AXS D8 Discover XRD diffractometer, equipped with a CuKα source, operated at 40 kV and 40 mA, along with a Lynxeye 1-dimensional detector, was employed for the analysis. Scans were done within the range of 3–75 °2θ, with a 0.05 °2θ step and a measuring time of 1 s/step by point. The Diffract-Eva software from Bruker, coupled with the PDF-2 mineralogical database from the International Centre for Diffraction Data (ICDD), was utilized to identify the crystalline phases.

#### 4.2.3 Physical properties

1) The determination of open porosity accessible to water followed the standard (EN 1936:2006), utilizing specimens with a side length of 50 mm. Each specimen was weighed and subsequently placed within a vacuum container where the pressure gradually decreased until it reached approximately 2.0 ± 0.7 kPa (15 ± 5 mm Hg). Subsequently, demineralized water was introduced slowly into the container until the specimens were fully immersed. The vacuum was maintained for an additional 24 h while submerged. Following this period, the pressure returned to atmospheric levels, and the specimens remained submerged in water under atmospheric pressure for over 24 h. After the elapsed time, each saturated specimen was weighed both in water and in the air.

The test was performed on non-exposed samples, as well as after exposure to 200, 400 and 600 °C followed by water cooling.

#### 4.2.4 Mechanical properties

1) Uniaxial compressive strength (σ_C_) was assessed in accordance with the EN 1926:2006 standard on 10 cubic specimens (50 mm side). The equipment used was a EL200 hydraulic press by PEGASIL with 1200 kN range capacity, accuracy of 0.01 kN and a load rate capacity of 1 ± 0.5 MPa/s. Load was applied perpendicular to the bedding planes.2) The measurement of hardness was conducted using a portable Leeb D EQUOTIP tester from Proceq, integrated with the wireless software platform EQUOTIP LIVE. The instrument underwent verification in accordance with the ISO16859–2:2015 normative. With a measurement capacity of 1000 HDL and an accuracy of 1 HLD, the instrument was employed to test the hardness of 10 specimens. Six measurements for each face were performed.
3) Measurements of the Ultrasonic pulse velocity (V_p_) according to the standard (EN 14579:2004) were carried out using a portable PUN-DIT PL200 PROCEQ. Non-destructive ultrasonic techniques were used to evaluate the conservation state of the limestones. A PUNDIT PL200 with 54 KHz transducers was used to calculate the speed of the longitudinal wave (V_p_) passing through the same stones before and after the test. Gel was used as coupled material. Three measurements were performed in the direction parallel to each specimen bedding plane. These tests were performed on non-exposed samples, as well as after exposure to 200, 400 and 600 °C followed by water cooling.

#### 4.2.5 Thermogravimetric analyses

1) Thermogravimetric analysis (TGA) was made using a volume of ∼0.2 cm^3^ of powder placed in platinum crucibles. The test was carried out using a thermobalance Netzsch STA449F3jupiter under Argon flow (60 mL/min) in the temperature range 40–1000 °C with a heating rate of 10 °C/min.

#### 4.2.6 Dimensioning methods – flooring and cladding

In addition, heat effect on stone slabs used for flooring and external cladding applications was analyzed in two case studies to assess stone slabs minimum recommended thickness (t) for external wall cladding and flooring applications. The methodology selected to dimension the stone slabs for cladding was based in a simple approach through Equation described by Cruz et al. [51] which account for permanent or dead load (self-weight) and live loads (foot traffic) and vibrations (Eq. (3)):

$$t=\sqrt{\frac{1.6\;1500.l.R_{L}}{R.w}}$$

where *l* represents the slabs length in mm; *w* is the slabs width in mm; *R*_*L*_ is the flexural rupture load in kN according to the table supplied by Cruz et al. and *R* is the rupture modulus in MPa [51].

The methodology selected for external wall cladding dimensioning was based upon Eurocode 1 (EN 1991:2002) and other relevant works publish in the field of natural stone [52,53]. The purpose of this assessment was to demonstrate how limestones cladding can be selected while accounting for fire effects. The minimum recommended thickness of stone slabs (t, in millimeters) was evaluated through Equation (4), proposed by Camposinhos and Lewis [52,54]:

$$t\gg L_{2}\sqrt{\frac{W_{Sd}}{\sigma_{Rd}}}$$

Where: W_Sd_ represents the wind load dynamic pressure (in kPa); L_2_ is the span dimension of the slab (in meters) and σ_Rd_ is the flexural strength (in MPa).

Thickness calculations were made for three distinct slab spans (L_2_) typically chosen for ventilated stone facades. A lateral average wind load (W_Sd_) with a dynamic pressure of 1 kPa was chosen. This wind dynamic pressure corresponds to the average value typically used for a 35-m-high building (approximately 12 floors) located in urban areas. For additional wind load data, Eurocode 1 provides further information.

In both case studies (wall cladding and flooring) the calculations considered the following scenarios.

• Slab thickness based on the reference value of flexural strength before heat treatment.
• Slab thickness based on the calculated mean value flexural strength after heat treatment.

## 5 Results and discussion

The performance of the two limestones, LL and GAS, under high temperatures followed by rapid cooling, reveals significant differences and underlying mechanisms driving their deterioration. This section provides insights into the macroscopic and microscopic alterations, mechanical strength degradation, porosity changes, hardness, and ultrasonic pulse velocity variations, all of which are crucial for understanding their behaviour under fire conditions.

### 5.1 Petrographic and mineralogical composition

**Fig. 4:**
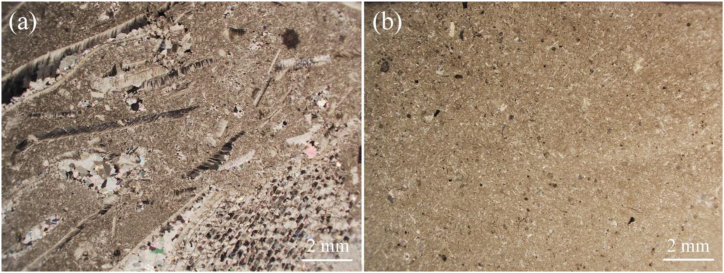
(a) LL limestone (biosparite) and GAS (biomicrite).

The petrographic analysis depicted in Fig. 4 allowed to verify that LL is a recrystallized limestone with recognizable depositional grain-supported texture. It has 50 % vol. of spiritic cement with about 48 % vol. of the thin section consisting of bioclasts association. The estimated porosity is around 2 %. Given the high content of sparite and the presence of bioclasts, the rock is considered as biosparite [55]. XRD analyses of LL evidenced 97 % of calcite, 1.8 % of plagioclase and 1.2 % of quartz.

GAS is a carbonate stone with mud-supported depositional texture. It is composed by a micritic matrix (>92 % vol.). Quartz is present up 2 %. The organic matter is present up to 6 % vol. Due this textural features and the high content of bioclasts, GAS classified as biomicrite [55]. XRD analyses shown the presence of calcite (99 %) and quartz (1 %).

### 5.2 Macroscopic features – color changes and deterioration paterns

The limestone specimens were initially examined in their original state before the thermal cycle, and 24 h later, following storage at 20 ± 1 °C and 50 % relative humidity (Fig. 2a and (b)). Among the two limestones, there is a visible change in color due to exposure to high temperatures, even before rapid cooling by water.

**Fig. 5:**
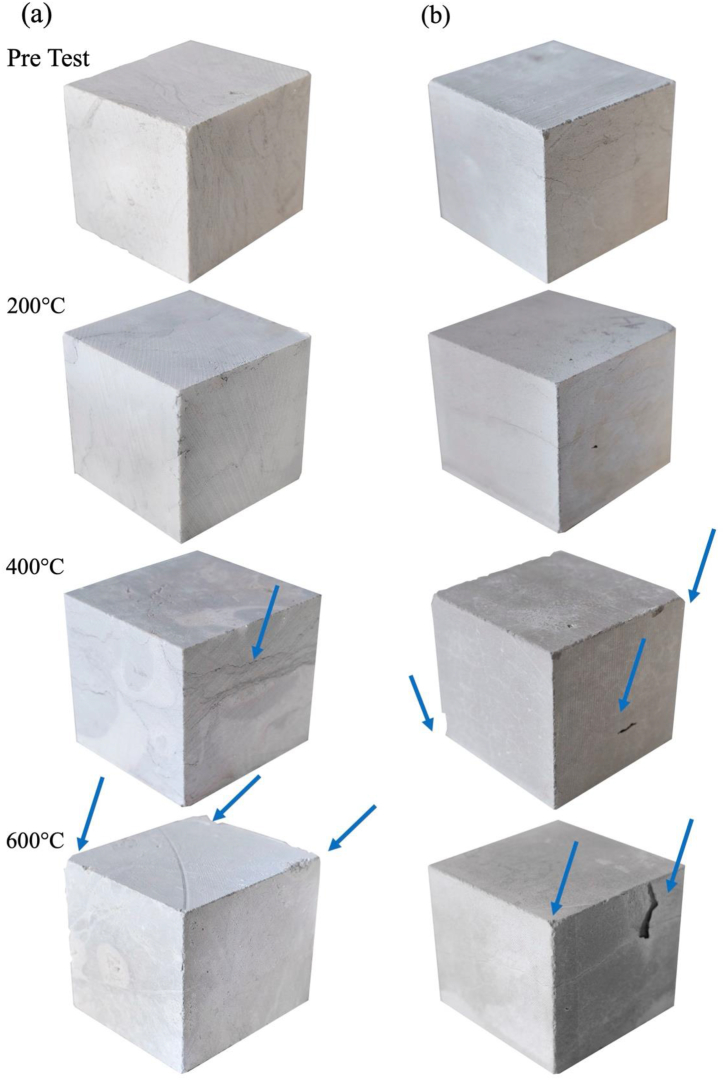
Qualitative features of thermally altered samples compared with pre-tested specimens of 5 cm side cubes. Macroscopic features of LL **(a)** and GAS **(b)** depicting the effect of each heating – cooling in water: at initial state before heating; after heating at 200 °C; after heating at 400 °C and after heating at 600 °C and cooling in water. A noticeable color change is detected for both stones from 400 °C. Spalling and material loss were more prominent from 400 °C in GAS specimens (blue arrows). Stylolites enhancement was noticeable from 400 °C in LL. (For interpretation of the references to color in this figure legend, the reader is referred to the Web version of this article.)

From 400 °C conditions, both limestones, but especially GAS, appear to show the first signs of deterioration (Fig. 4), from small mass loss by superficial crumbling, to decohesion of the material leading to partial disintegration after 600 °C (depicted by the blue arrows in Fig. 5). LL also depicted an enhancement of stylolites from 400 °C. No sign of superficial cracking was detected.

**Fig. 6:**
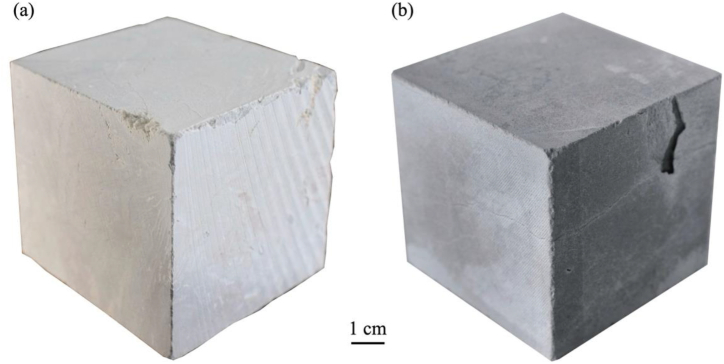
Qualitative features of thermally altered samples LL **(a)** and GAS **(b)** after heating at 600 °C and cooling in water. Spalling and material loss were more prominent at 600 °C in GAS specimens.

After the thermal cycle at 600 °C, deterioration increase in GAS with spalling and LL showed more decohesion and partial disintegration (Fig. 6). Still, both limestones allowed measurement of mechanical performance such as uniaxial compression strength.

The observed deterioration, progressing from the surface to the core of the samples, can be attributed to the thermal shock process that occurred during the water-cooling stage, which was intensified by the increased open porosity and deepened by pre-existing thermally induced micro-cracks.

Both LL and GAS limestones exhibit visible changes in color when exposed to temperatures starting from 400 °C, with GAS showing more pronounced alterations. This is primarily due to the thermal decomposition and oxidation of minerals like iron oxides and iron sulphides, as well as the transformation of organic matter into coal. As reported in previous researches for the same temperature range, the color changes serve as an early indicator of thermal ageing, which is crucial for assessing fire-affected structures [13,17,26,56]. The deterioration patterns of the two limestones diverge at higher temperatures.

i) LL: from 400 °C it shows enhancement of stylolites and by 600 °C, significant decohesion and partial disintegration are evident. The increase in intergranular and transgranular porosity suggests a substantial worsening of its structural integrity.
ii) GAS: it exhibits more severe spalling and material loss starting from 400 °C. The presence of higher open porosity initially facilitates gas release (H_2_O and CO_2_), but by 600 °C, extensive crack enlargement critically affects its mechanical performance.

Variations in color between the reference and thermally aged limestones were quantified by color distance (ΔE*) calculated using Equation (2). A detailed examination of the color alterations resulting from the heat treatment is depicted in Fig. 5.

**Fig. 7:**
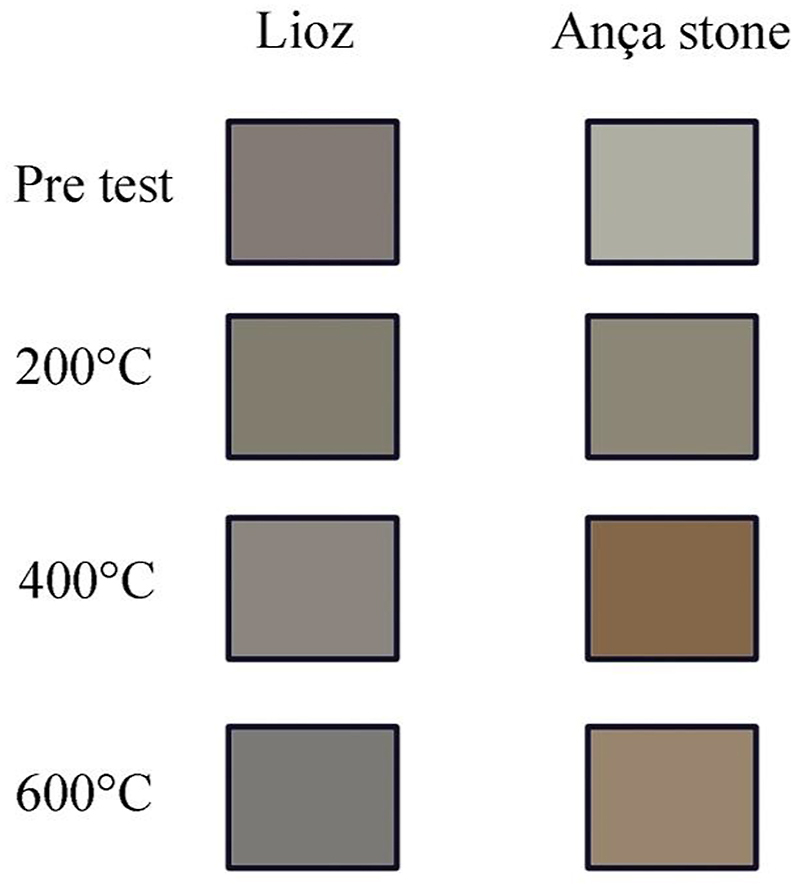
Color change digital reconstruction depicting the effect of each heating – cooling in water: at initial state before heating; after heating at 200 °C; after heating at 400 °C and after heating at 600 ^◦^C– cooling in water. A noticeable color change was detected for GAS limestone from 400 °C turning from grey to brown hues. (For interpretation of the references to color in this figure legend, the reader is referred to the Web version of this article.)

Natural stones undergo color changes to hues of pink, red, or black when exposed to fire damage, primarily attributed to the thermal decomposition and oxidation of the minerals composing the stones, even in very low percentage oxidation (i.e. iron oxides and/or iron sulphides as visible in Fig. 5, Fig. 6, Fig. 7 and also bioclasts [13,17,26]. Furthermore, organic matter which turns into coal also play a fundamental role in color changes [13,17,26].

Results shown in Table 2 and Fig. 7 stressed that there is a color change (ΔE*) in both limestones after being subjected to the different heating temperatures followed by water cooling. As seen in Fig. 5, Fig. 6, Fig. 7, which the latter is a digital reconstruction of (ΔE*) to better represents the color modification, it is difficult to visually identify any color change until 200 °C however, when the temperature raises from 400 °C and until 600 °C changes are clearly visible as stressed by previous works [56].

**Table 2:** Numeric data of color coordinates in CIE L*a*b system in pre-test and heating-water cooling samples at three temperature steps.

Stone type	LL				GAS			
Test conditions	Pre-test	200	400	600	Pre-test	200	400	600
L*	52	55	56	51	71	56	46	57
ΔL*		3	4	−1		−15	−25	−14
a*	2	0	1	0	−2	0	9	5
Δa*		−2	−1	−2		2	11	7
b*	4	8	4	2	6	10	22	16
Δ b*		4	0	−2		4	16	10
ΔE*		5	4	3		16	32	19

All the recorded color distances are over the color perceptibility threshold (ΔE* ≥ 3), [57]. The total color differences, represented by ΔE*, confirm the macroscopic observations indicating that GAS exhibits the most noticeable color change to the human eye (Table 1). It's crucial to note that the minimum threshold for color perception by observers is still under investigation, which may pose particular challenges for rocks with heterogeneous coloring [58].

As displayed in Table 2, Fig. 5, Fig. 6, Fig. 7, the color drop in L* values after heating is commonly associated with the onset of calcite thermal metamorphism, yet it varies even among the two limestones composed of 97–99 % calcite.

The following observations are consistent in both tested limestones.

i) An increase in temperature resulted in a decrease in the parameter and b* leading to shift to bluish hues in both limestones (+b* toward yellow and −b* toward blue);
ii) The decomposition of calcite to lime resulted immediately in a whitening effect on the stone (particularly after heating at 600 °C), however once the stones were cooled in water (20 °C) a significant increase in the parameter L* was detected.

Chromaticity coordinate a* show distinct tendency in both limestones. While LL revealed a small negative shift towards red hues, GAS displayed a small positive shift towards green. Color of both limestones, before and after heating-cooling, may be explained by the composition of clay minerals on the unheated stones and after by oxidation of the minerals that are present [7,26].

The reported changes, surpassing noticeable thresholds, underscore the challenges in accurately assessing color perception, especially in rocks with heterogeneous composition and coloring. The primary cause is ascribed to the thermal decomposition and oxidation of the minerals present in the stones, even at minimal levels of oxidation (e.g., iron oxides and/or iron sulphides) along with bioclasts. Additionally, the transformation of organic matter into coal also significantly contributes to color alterations.

### 5.3 Microscopic observations

**Fig. 8:**
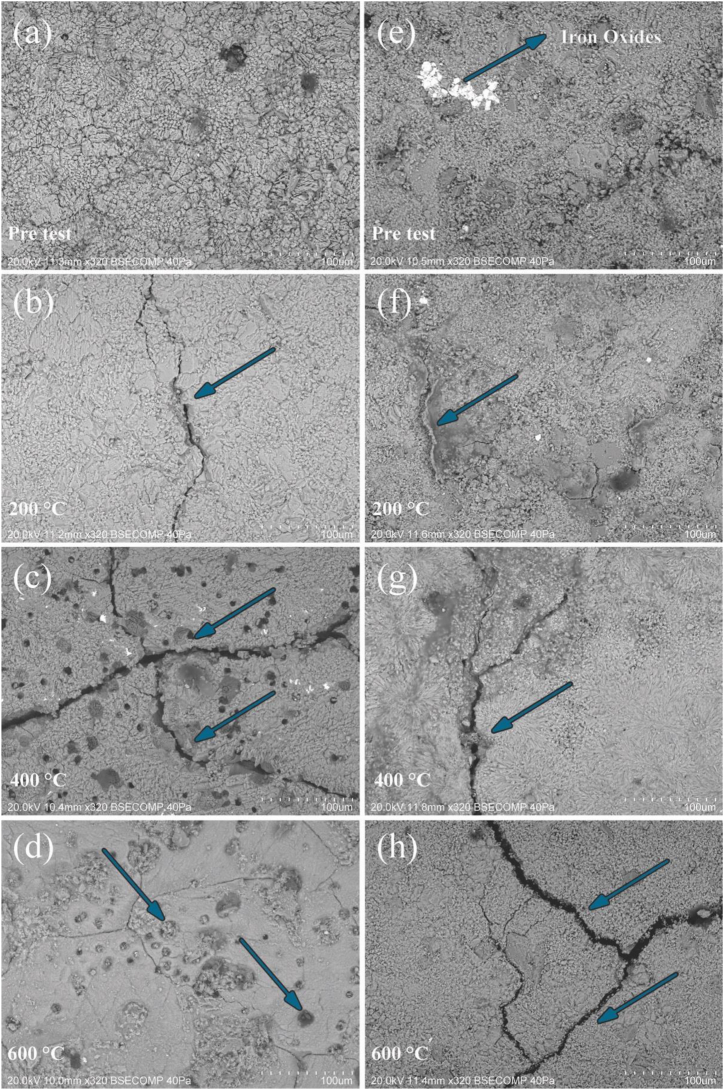
Microscopic features of LL **(a, b, c, d)** and GAS **(e, f, g, h)** depicting the effect of each heating – cooling cycle in water: at initial state before heating; after heating at 200 °C; after heating at 400 °C and after heating at 600 ^◦^C– cooling in water. A noticeable increase in the number of intra and inter microcracks, highlighted with blue arrows, was detected in both stones from 400 °C. Also, intragranular porosity (“holes” formation) started from 400 °C in LL and increased in dimension at 600 °C due to the loss of the volatiles (H_2_O and CO_2_) through the compact solid fraction of the stone (highlighted with blue arrows). In GAS stone, Intragranular increase porosity it is not so evident due to the higher open porosity which facilitates the release of gases as the temperature increases. (For interpretation of the references to color in this figure legend, the reader is referred to the Web version of this article.)

Scanning electron observations made on reference and heating-cooled specimens after all studied temperatures, were compared and revealed critical insights (Fig. 8).

i) LL: Exhibits substantial intergranular and transgranular cracking linked to the presence of stilolytes, with pore dimensions increasing up to 20 μm at 600 °C. The compactness of calcite crystals decreases significantly, leading to a notable reduction in structural integrity (Fig. 8c and d).
ii) Compactness and Cracking: LL limestone showed distinct compact and granular areas with intergranular microcracks ranging from ∼0.1 to 2 μm (Fig. 8a). As heating temperature increased, these microcracks expanded (Fig. 8c).
iii) Emergence of Pores: At 400 °C, intergranular pores started to appear in LL, increasing in size with temperature, reaching ∼10–20 μm at 600 °C due to the release of H_2_O and CO_2_ (Fig. 8b, c and 8d).
iv) GAS: Although initially more compact with fewer microcracks and contained agglomerates of iron oxides, explaining color changes to brown and darker hues, GAS develops significant intergranular microcracks with dimensions comparable to LL at higher temperatures (Fig. 8e, g and 8f). However, due to its higher initial porosity, the impact on mechanical performance is somewhat mitigated.
v) Temperature Effect on Cracks: After heating-cooling at 200 °C, intergranular microcracks of 0.1–1 μm appeared, increasing to ∼10 μm at 400 °C and ∼10–20 μm at 600 °C (Fig. 8f, g and 8h).
vi) Impact on Mechanical Performance: While the 600 °C heating-cooling cycle had a more modest impact on the pore structure of GAS, the significant enlargement of cracks critically affected its mechanical performance (Fig. 8h).

The extent of fire damage to a rock is influenced by its thermal conductivity, heat absorption, and intrinsic factors such as porosity and grain size [7,11,26,59]. Several stone materials with different minerals and microstructures could present different variations in physical and mechanical properties with increasing temperature. Simmilarly to the effect reported in previous studies, examination of the SEM images showed that the specimens experienced significant changes with increasing temperature, so that some micro-cracks appear on the surface of specimens from 400 °C after water cooling. These fractures preferentially follow the stilolythes in both limestones where the stone structure was highly weakened due to loss water after heating [7,11,26,59].

### 5.4 Uniaxial compressive strength

The data set present in Table 3 show uniaxial compressive strength results for both limestones (LL and GAS) in pre-test and heated specimens at three temperature steps. The uniaxial compressive strength results highlight different deterioration mechanisms.

i) LL: Experiences a minor strength reduction up to 400 °C but shows a dramatic 33 % decrease at 600 °C. This is attributed to the significant increase in intergranular porosity and microcracks, leading to substantial structural weakening. Before reaching 600 °C, the drop in mechanical strength in LL was still within the standard variation range (8–10 %) for natural stone [60].
ii) GAS: Exhibited a more gradual reduction in strength, with a 22 % decrease at 400 °C and a 23 % decrease at 600 °C. Although the total percentage drop is less than LL, the initial higher strength and porosity of GAS play a role in its overall resilience. GAS compressive strength results show a similar trend than LL with a small difference, uniaxial compression strength decreased 22 % after 400 °C heating-cooling cycle and almost kept constant after 600 °C (23 %). Authors believe that this effect is strictly linked to the thermal-shock effect when specimens are cooled. Like stated by the authors in previous research works, and as explored by other researchers, this decay can be explained by the effect of thermal expansions/contractions of LL and GAS minerals originated during heating and cooling stages [13,17,22,59].

**Table 3:** Numeric data of uniaxial compression strength data in pre-test and thermal aged specimens at three temperature steps.

Stone type	Test method	Pre-test	After heating- cooling in water		
			200 °C	400 °C	600 °C
LL	Compression strength - σ_C_ [MPa]	113 ± 2	112 ± 5	107 ± 9	76 ± 10
		vc = 0.2 %	vc = 5 %	vc = 8 %	vc = 13 %
	Δ [%]	–	1	6	33
GAS	Compression strength - σ_C_ [MPa]	150 ± 8	145 ± 15	118 ± 15	115 ± 9
		vc = 5 %	vc = 10 %	vc = 13 %	vc = 8 %
	Δ [%]	–	4	22	23

vc = variation coefficient [%].

As exhibited in Fig. 8, heat and subsequent shock cooling by water caused an increase in the number and dimension of microfractures and in intra and inter grain porosity. As reported by other authors, a noticeable increase in intergranular pores after 400 °C can significantly impacted the limestone's mechanical performance, particularly its uniaxial compression strength [61,62]. Despite this deterioration, mechanical strength remains measurable even after thermal aged at 600 °C, indicating complex decay kinetics influenced by initial mechanical strength and porosity of the stones.

### 5.5 Open porosity

**Fig. 9:**
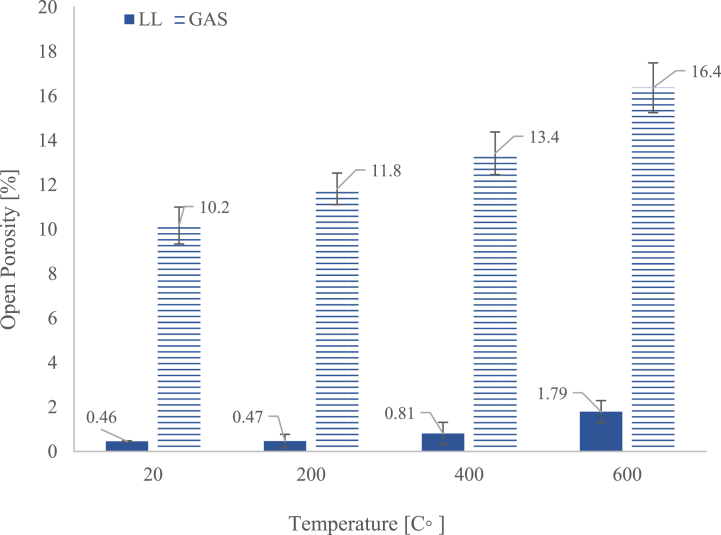
Average open porosity results in pre-tested (20 °C) and after the thermal cycle followed by cooling at temperatures of 200 °C, 400 °C and 600 °C. A correlation between temperature and porosity is proposed.

Fig. 9 exhibits the data of the open porosity (%) measured along the temperature steps (after cooling in water). Overall, akin to the findings observed in uniaxial compression strength, there exists a direct correlation between temperature and open porosity. Porosity analysis indicates a substantial increase in open porosity with rising temperatures.

i) LL: Displays an almost threefold increase in porosity from pre-test to 600 °C, driven by the formation of new pores due to the loss of volatiles (H_2_O and CO_2_) - LL-pre-test = 0.46 ± 0.02 %, LL-200 °C = 0.47 ± 0.3 %, LL-400 °C = 0.81 ± 0.5 % and LL-600 °C = 1.79 ± 0.5 %. Globally, LL open porosity increased ∼289 %.
ii) GAS: Shows a 70 % increase in porosity, which, while less drastic than LL, still represents a significant structural change - GAS-pre-test = 10.2 ± 0.8 %, GAS-200 °C 11.8 ± 0.7 %, GAS-400 °C = 13.4 ± 1.0 % and GAS-600 °C = 16.4 ± 1.1 %. The pre-existing higher porosity facilitates gas release but also contributes to further weakening when cracks enlarge.

Porosity changes in LL can by explained due to due to the loss of the volatiles (constitutional H_2_O and CO_2_) through the compact solid fraction of the stone leading to new “holes “formation”. Pre-tested LL has substantially low open porosity compared to GAS. In GAS, intragranular increase porosity it is not so evident due to the higher open porosity which facilitates the release of gases as the temperature increases. However, the porosity increases of 70 % is also caused by the crack enlargement which allowed space for water, as identify in Fig. 8.

The increase in stones porosity when subjected to simulated fire conditions has been reported in previous works [8,12,13,20]. The degree of porosity alteration is contingent upon the inherent "compactness" of the stone; denser stones exhibit more pronounced changes in porosity at elevated temperatures compared to those with lower cementation levels [12,61]. The alteration of mechanical properties in natural stones due to increased porosity induced by temperature variations is a notable aspect. The rise in porosity elucidates the concurrent decrease in compressive strength, displaying the weakened lattice structure.

Microstructure analysis depicted in Fig. 8., uncovered significant changes, including the propagation of microcracks and pore enlargement, particularly pronounced at higher temperatures. While GAS limestone initially exhibited a denser/compact structure, significant cracking enlargement impacts its mechanical performance. This, coupled with a direct relationship between temperature and open porosity increase, highlights the critical role of temperature in altering limestone properties, with implications for mechanical durability and long-term performance.

### 5.6 Leeb D hardness

**Fig. 10:**
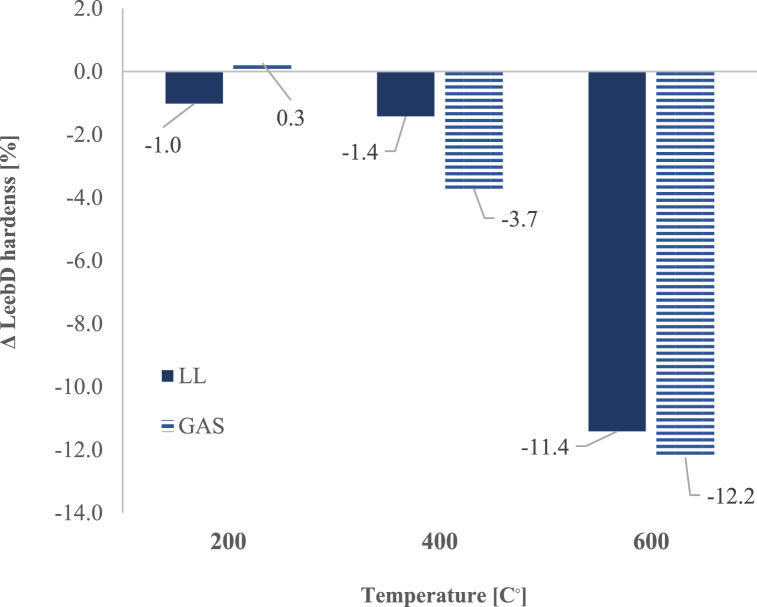
Variation of Leeb D hardness (%) as function of temperature until 600 °C, compared to initial results assessed at 20 °C.

Variation of Leeb D hardness (%) as function of heating-cooling until 600 °C, was compared to initial results assessed at 20 °C in Fig. 10. Leeb D hardness and ultrasonic pulse velocity measurements correlate with the observed mechanical degradation.

Both limestones show a reduction in hardness at 600 °C, with GAS experiencing a slightly greater decline (12.2 %) compared to LL (11.4 %) (Table 4). Results seem to indicate that Leeb D hardness does not change critically with temperature until 600 °C. Variations in composition and structural characteristics among limestone types result in distinct changes in hardness. Particularly, the hardness values shifted at 600 °C for LL (Δ = −11.4 %) and GAS (Δ = −12.2 %) specimens. This is since, at higher thermal shock fractures, microcracks and distortion are more severe.

**Table 4:** Average Leeb D hardness (%) as function of heating-cooling cycles until 600 °C.

Stone type/Temperature	Pre-Test	200 °C	Pre-Test	400 °C	Pre-Test	600 °C
LL	668.0 ± 27.4	662 ± 34.3	660.0 ± 14.3	650.6 ± 16.1	658.8 ± 26.2	583.6 ± 31.2
vc [%]	4	5	2	5	4	5
GAS	590.2 ± 5.2	591.71 ± 16.0	584.8 ± 8.7	563.0 ± 30.6	588.0 ± 9.7	516.0 ± 25.3
vc [%]	1	3	1	5	2	5

vc = variation coefficient [%].

Among the two natural stones examined, GAS experienced the most significant reduction in hardness. For instance, the hardness of the GAS specimens decreases from −3.7 % after 400 °C and −12.2 % after 600 °C.

### 5.7 Ultrasonic pulse velocity

**Fig. 11:**
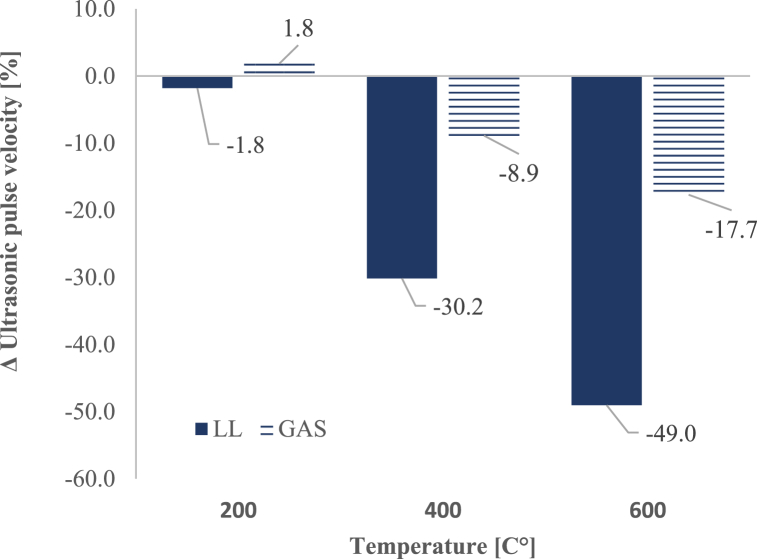
Ultrasonic pulse velocity changes (%) as function of temperature until 600 °C compared to initial results assessed at 20 °C.

Evolution of ultrasonic pulse velocity (m/s) as function of temperature until 600 °C, compared to initial results assessed at 20 °C is shown in Table 5 and Fig. 11 depicts Ultrasonic pulse velocity changes (%) as function of temperature. Likewise the results presented by other authors, from 400 °C and above, ultrasonic pulse velocity of samples progressively decreases, revealing a progressive damage to the stone by temperature [61,62]. LL presents a more significant decrease in ultrasonic pulse velocity, with 49 % reduction at 600 °C, indicative of severe internal damage. GAS also shows a decrease, but less pronounced (17.7 %), aligning with its higher initial porosity and different crack propagation behavior.

**Table 5:** Average ultrasonic pulse velocity (m/s) as function of heating-cooling cycles until 600 °C.

Stone type/Temperature	Pre-Test	200 °C	Pre-Test	400 °C	Pre-Test	600 °C
LL	5016.4 ± 423.6	4925,7 ± 400.3	6020.5 ± 97.0	4205.2 ± 334.5	5801.2 ± 119.3	4164.8 ± 230.6
vc [%]	8	8	2	8	2	11
GAS	4234.9 ± 11.5	4313.1 ± 85.1	4252.7 ± 64.6	3875.3 ± 114.0	5057.9 ± 216.4	516.0 ± 25.3
vc [%]	3	2	2	3	4	6

vc = variation coefficient [%].

In LL, after 400 °C heating-cooling cycle, there is a significant 30.2 % decline in ultrasonic pulse velocity, whereas GAS limestone experienced reductions of no more than 8.9 %. Furthermore, the evolution of LL presents two distinct phases: an irrelevant small drop after 200 °C (1.8 %) reaching a relevant drop at 400 °C (30.2 %), followed by a second phase characterized by a decrease of 49 %. Likewise, in GAS, ultrasonic pulse velocity changes in a lower level but with a similar trend: 1.8 % in absolute value after 200 °C, 8.9 % after 400 °C and 17.7 % after 600 °C heating-cooling cycle.

Ultrasonic pulse velocity is influenced by the type of damage as well as the presence of stone pores and fissures simultaneously [17,63]. Therefore, this approach enables not only the determination of the ultrasonic pulse velocity but also the evaluation of the overall damage incurred. A decline in the ultrasonic pulse velocity of limestones will inherently correspond to an overall reduction in compression strength and mechanical performance.

Thermal shock induced reductions in hardness and ultrasonic pulse velocity are linked to the lowering of compression strength and of overall mechanical performance. These changes, related to the mass loss can be attributed to various physical transformations such as the evaporation of internal water, the decomposition of certain minerals such as calcite, and a small amount of rock chip flaking and spalling [17] [12,18,21]. Additionally, the calcium carbonate decomposition releasing carbon dioxide gas and leaving behind calcium oxide and the formation of numerous fractures under thermal shock, collectively contribute to changes in limestones volume. This emphasizes the need for comprehensive understanding of temperature effects on limestones. Such insights are crucial for structural design considerations, particularly in ensuring adequate load support and durability in limestone-based structures.

### 5.8 Thermo-gravimetry analysis

Thermo-gravimetry analysis (TGA) provides meaningful observations into the thermal behavior of studied limestones by measuring changes in mass as a function of temperature. This analytical technique helps to identify the decomposition temperatures of various mineral phases present in limestone, such as calcite, and to assess their thermal stability. By understanding how limestones react to heat is possible to make informed decisions regarding their processing, utilization, and performance in various applications, including as construction materials. Additionally, thermal gravimetry analysis can aid in the characterization of impurities, organic content, and thermal decomposition pathways, contributing to a comprehensive understanding of limestone properties and behavior under the studied thermal conditions.

**Fig. 12:**
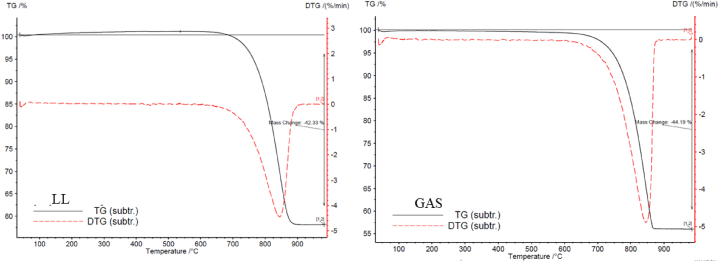
Curves of thermal decomposition obtained through TGA analysis.

TGA results show that the heating cycles carried out in the muffle furnace did not allow for complete thermal metamorphism of the LL and GAS limestones since the tests were conducted only up to 600 °C (Fig. 12). Both LL and GAS show complete thermal metamorphism around 900 °C, with significant mass loss indicating the breakdown of calcite into calcium oxide and carbon dioxide. The partial thermal metamorphism observed up to 600 °C correlates with the mechanical and structural changes, emphasizing the role of mineral decomposition in overall deterioration.

As can be observed in Fig. 12 thermal metamorphism refers to the process by which calcite, a common mineral found in the analyzed limestone, undergoes a decomposition upon heating. This endothermic reaction occurs when calcite is heated to temperatures typically above 550–600 °C, causing it to break down into calcium oxide (CaO) and carbon dioxide (CO_2_). In the studied limestones both the thermal decomposition curves show complete thermal metamorphism at around 900 °C by a considerable mass loss of 42.33 % in LL and 44.19 % in GAS. After 900 °C, both the thermal decomposition curve of Fig. 12 does not show any modification due to the stability of CaO.

The results from Fig. 12 also show, for both limestones studied, a loss of mass due to retained water (∼40 °C) and loss of hydration water at ∼ 120 °C (both endothermic reactions). Loss mass is highlighted by two negative peaks in correspondence of the above-mentioned temperatures. No other chemical transitions were detected in the TGA.

### 5.9 Impact on heritage conservation practices

The research on the thermal ageing of LL and GAS limestones offers insights that can enhance the protection of heritage buildings from fire damage. By applying these data and findings to conservation practices, modern and historic structures can be preserved. It's essential to assess the fire risk for each heritage building looking at factors such as the building's location, the materials used in its construction, and the surrounding environment. The study can be relevant for sites including LL or GAS limestone, providing a clear understanding of how these materials respond to heat, but the approach and preventive measures can be developed for any site. This might involve managing vegetation, creating firebreaks, and raising public awareness about fire risks [64,65]. Preservation and restoration techniques are also critical because when choosing protective/conservation treatments and reinforced systems [66]. In case of cultural heritage is crucial to select those that can shield the stone from heat without compromising its appearance or durability.

Emergency and response plans are another vital aspect. Modern fire detection and suppression systems should be installed, capable of detecting early signs of fire without causing damage to the building. Regular updates to emergency response plans and training for staff and local firefighters on handling heritage sites with care are equally important.

Monitoring and maintenance are ongoing needs. Regular inspections for signs of thermal damage and prompt addressing of any issues can prevent further deterioration. Maintenance programs should include cleaning, reapplying protective coatings, and performing necessary repairs to ensure the limestone remains in good condition.

Supporting ongoing research into the thermal performance of different heritage materials is also beneficial. Educational programs can train conservators, architects, and engineers on best practices for preserving limestone against fire damage, further enhancing the overall preservation efforts.

Collaboration with fire safety experts and heritage conservation specialists can help integrate the latest research into practical conservation strategies. Advocating for policies that incorporate your findings can improve the fire resilience of heritage buildings on a broader scale. For example, if your study indicates that GAS limestone is more vulnerable to heat compared to LL limestone, it would be prudent to prioritize protective measures for buildings made of GAS limestone. Additionally, using LL limestone for restoration in high-risk areas can provide better resilience. By applying these findings comprehensively, we can significantly enhance the protection and preservation of our precious heritage buildings, ensuring they withstand fire risks and remain intact for future generations.

### 5.10 Impact of heat through simulated fire conditions on stone slabs dimension – a case study

Designing and evaluating the thickness of a stone slab for external wall cladding and flooring applications is crucial and directly influenced by the anticipated level of loads acting on the stone element. A comprehensive understanding of these load types informs the stone slab thickness, reinforcement, anchoring, and joint design (EN 1991:2002) [52].

a) Permanent or dead load: This refers to the weight of the stone slab itself, as well as any other permanent fixtures or materials attached to it. Dead load includes the self-weight of the stone, adhesive, and any additional layers (such as insulation or waterproofing).
b) Transient or live load: live load which represent the dynamic forces imposed on the stone slab due to human activity, furniture, equipment, or other movable objects. For flooring, this includes foot traffic, furniture placement, and occasional loads. In the case of facades, live load can result from wind-induced vibrations, maintenance personnel, or temporary equipment (e.g., scaffolding during construction).
c) Wind load: facades are particularly susceptible to wind pressure. Wind load varies based on the building's location, height, shape, and exposure. It exerts both inward and outward forces on the stone panels. The design must account for wind speed, direction, and the building's aerodynamic characteristics.
d) Thermal expansion and contraction: temperature fluctuations cause stone panels to expand and contract. This movement can lead to stress and affect the slab's stability. Proper expansion joints and allowances for thermal movement are essential.
e) Seismic load: in seismically active regions, stone facades and flooring must withstand ground motion. Seismic forces can cause lateral movement, shear, and overturning. Reinforcement and anchoring systems are critical to prevent failure during earthquakes.
f) Impact load: stone slabs may experience sudden impacts, such as heavy objects falling or accidental collisions. Impact loads can cause localized damage or fractures. Impact-resistant design and material selection are crucial.
g) Moisture and chemical exposure: stone panels in outdoor applications face rain, humidity, and chemical exposure. Moisture can lead to efflorescence, staining, and freeze-thaw cycles. Proper waterproofing and chemical-resistant treatments are necessary.
h) Vibration and vibrations: vibrations from nearby machinery, transportation, or even music can affect stone panels. Excessive vibrations may lead to fatigue and cracking. Damping systems and resilient mounts can mitigate vibration effects.
i) Foundation settlement and subsidence: uneven settling of the building foundation can cause differential movement in stone slabs. Subsidence due to soil conditions also impacts stability. Proper foundation design and monitoring are essential.
j) Maintenance loads: stone panels require periodic maintenance, such as cleaning, sealing, and repairs. Access for maintenance personnel and equipment must be considered during design for both wall cladding and flooring.

In general thicker slabs offer increased durability and longevity, reducing the risk of cracking or damage over time [51]. On the other hand for example, in areas with lower foot traffic or in lower buildings with less prominent wind action, thinner slabs (25–35 mm) may be sufficient while still providing the desired aesthetic and functional qualities. Assessing the appropriate thickness involves considering various factors, including the type of stone, its inherent strength properties, the expected frequency and intensity of use, and any additional support or reinforcement measures required [51,67].

Ultimately, the proper design and assessment of stone slab thickness ensure not only the structural integrity and safety but also its long-term performance and aesthetic appeal, contributing to a functional and visually pleasing environment.

#### 5.10.1 Stone slabs for flooring

The primary purpose of the case study was to showcase how, initially, the thickness of stone panels for floor applications may fluctuate when considering the impact of fire on the mechanical strength of limestones. Stone slabs thickness t (in mm) was assessed from Eq. (3), proposed by Ref. [51] and results are shown in Table 6. It is essential to enhance that the ratio between limestone's flexural and compression strength can vary depending on factors such as the specific type of limestone, its porosity, mineral composition, and structural characteristics. Typically, this ratio falls within a range of 10–20 % for most types of limestone and a ratio of 10 % was selected for this case study. However, it's important to note that, this ratio is not universally consistent and may differ based on the geological properties and testing methods used [68,69],.

**Table 6:** Stone slab for floor thickness (mm) simulation according to the decay levels achieved in the heating-cooling cycles for LL and GAS.

Stone type	Flexural Strength [MPa]	t (mm) and R_L1_ = 0,75 kN	t (mm) and R_L2_ = 3,5 kN	t (mm) and R_L3_ = 6 kN
Pre-test LL	11.5 ± 2.0	13	27	27
LL 200 °C	11.2 ± 1.8	13	27	27
LL 400 °C	10.7 ± 1.8	13	28	28
LL 600 °C	7.6 ± 1.9	15	33	33
Pre-test GAS	15.0 ± 1.6	11	24	24
GAS 200 °C	13.1 ± 1.0	12	25	25
GAS 400 °C	10.6 ± 1.2	13	28	28
GAS 600 °C	10.4 ± 1.8	13	28	28

R_L1_ = Slabs laid on mortar or concrete for exclusive pedestrian use; R_L2_ = Slabs for pedestrian areas and bike lanes, gardens, and balconies; R_L3_ = Slabs for areas with occasional access by light vehicles and motorcycles. Driveway entrances.

As the mechanical strength of LL and GAS limestones is more affected by the high-temperature effect, higher will be its recommended thickness. The more the limestone's mechanical strength is affected by the high temperature, the greater its recommended thickness should be to adequately support the loads and intended use conditions. The potential lack of analysis on the mechanical decay of limestones caused by fire underscores a critical gap in understanding the full extent of damage and deterioration in stone structures. Without thorough examination and assessment of how fire impacts the mechanical properties of limestone, there is a risk of overlooking crucial factors that could affect the structural integrity and safety of buildings and monuments. Addressing this gap through comprehensive research and analysis is essential for developing effective strategies to mitigate the long-term effects of fire on limestone structures and ensure their resilience and longevity.

#### 5.10.2 Stone slabs for external wall cladding

Stone cladding projects typically commence with the client's or architect's vision. Drawings then make their way to the project engineering section, gradually evolving into final documents accompanied by a budget estimate. Surprisingly, neither of these stages often possesses substantial knowledge about stone performance—specifically related to the chosen stone materials, facade type, environmental conditions, or other factors impacting stone durability.

When stones are sold for cladding applications, they are often prescribed under the assumption that they won't encounter chemically or physically demanding situations. However, once stone slabs find their place in diverse environments, they become vulnerable to an array of stresses. As a reference for stone cladding applications, technical specifications and details concerning stones dimensioning and physical-mechanical recommended values may be found in relative few works considering the relative huge amount of different stone types settled in building facades [[52], [53], [70],^59^].

In this case study, three different stone slab spans were selected, based upon current market requests. A wind pressure of 3500 Pa was selected as an example of a 15-floor building. Stone slabs thickness t (in mm) was assessed from Eq. (5), and results are shown in Table 7 [[52], [53], [70]].

**Table 7:** Stone slab for external wall (mm) simulation according to the decay levels achieved in the heating-cooling cycles for LL and GAS.

Stone type	Flexural Strength [MPa]	Span (m)		
		L2 = 0.6	L2 = 1.2	L2 = 1.6
Pre-test LL	11.5	14	21	28
LL 200 °C	11.2	14	21	28
LL 400 °C	10,7	14	22	29
LL 600 °C	7,6	17	26	34
Pre-test GAS	15,0	12	18	24
GAS 200 °C	13,1	13	20	26
GAS 400 °C	10,6	15	22	29
GAS 600 °C	10,4	15	22	29

When it comes to LL and GAS limestones, it possible to detect that their mechanical strength is significantly impacted by high temperatures. Consequently, the recommended thickness should be adjusted accordingly. Essentially, the greater the influence of temperature on the limestone's mechanical strength, the thicker the slab should be to adequately withstand loads and fulfill its intended purpose.

It's crucial to emphasize that this practical case study serves as a simplified demonstration, focusing solely on the impact of wind on the facade. A comprehensive facade project requires a detailed analysis of all relevant factors and the establishment of safety coefficients. These coefficients are likely to adjust the reference thickness value presented here (Table 7), ensuring the structural integrity and safety of the facade design.

## 6 Conclusions

This study investigated the impact of high-temperature exposure followed by water cooling on two Portuguese limestones used as building materials for several purposes. Initially, experiments were carried out to uncover the primary mechanisms of alteration, including thermal-chemical transformations and physical changes. Subsequently, tests were conducted on specimens subjected to three different heating-cooling cycles at three temperatures to analyze changes in microstructure and physical-mechanical properties over time. The key findings suggest that while these limestones can be significantly impacted by exposure to high temperatures, their behaviors may vary as follows.

1. The first key finding was the noticeable color changes observed, particularly in the GAS limestone, as temperatures increase. These changes, surpassing noticeable thresholds, underscore the challenges in accurately assessing color perception, especially in rocks with heterogeneous composition and coloring. The primary cause is ascribed to the thermal decomposition and oxidation of the minerals present in the stones, even at minimal levels of oxidation (e.g., iron oxides and/or iron sulphides) along with bioclasts. Additionally, the transformation of organic matter into coal also significantly contributes to color alterations.
2. Moreover, structural alterations become evident with thermal exposure and along temperature increase, characterized by enhanced stylolites in LL and shifts to darker hues in GAS limestone. This structural transformation accompanies a decline in mechanical performance, attributed to intensified thermal shock causing microfractures and increased porosity. Despite this deterioration, mechanical strength remains measurable even after thermal treatment at 600 °C, indicating complex decay kinetics influenced by initial mechanical strength and porosity of the stones.
3. Microstructure analysis revealed significant changes, including the propagation of microcracks and pore enlargement, particularly pronounced at higher temperatures. While GAS limestone initially exhibited a denser/compact structure, significant cracking enlargement impacts its mechanical performance. This, coupled with a direct relationship between temperature and open porosity increase, highlights the critical role of temperature in altering limestone properties, with implications for mechanical durability and long-term performance.
4. Thermal shock induced reductions in hardness and ultrasonic pulse velocity are linked to the lowering of compression strength and of overall mechanical performance. These changes, related to the mass loss can be attributed to various physical transformations such as the evaporation of internal water, the decomposition of certain minerals such as calcite, and a small amount of rock chip flaking and spalling. Additionally, the calcium carbonate decomposition releasing carbon dioxide gas and leaving behind calcium oxide and the formation of numerous fractures under thermal shock, collectively contribute to changes in limestones volume.
5. As the mechanical strength of Lioz (LL) and Ançã stone (AS) limestones is significantly affected by high temperatures, it is crucial to recommend increased thickness for these materials to ensure they can support loads and intended use conditions adequately. The absence of comprehensive analysis on the mechanical decay of limestones due to fire highlights a critical gap in understanding the extent of damage and deterioration in stone structures. Without thorough examination, important factors affecting the structural integrity and safety of buildings and monuments may be overlooked. Addressing this gap through detailed research and analysis is essential for developing strategies to mitigate the long-term effects of fire on limestone structures, ensuring their resilience and longevity.

This emphasizes the need for comprehensive understanding of temperature effects on limestones. Such insights are crucial for structural design considerations, particularly in ensuring adequate load support and durability in limestone-based structures.

In final analysis, stones with higher toughness and compression strength, such as GAS, exhibited reduced damage at high temperatures due to their enhanced resistance to fracturing under stress. As limestone's mechanical strength decreases under high temperatures, it's advisable to increase its thickness to ensure sufficient support for loads and intended conditions of use. The deficiency of analysis on limestone's mechanical decay from fire reveals a significant knowledge gap regarding the complete extent of damage and deterioration in stone heritage structures.

## Funding

Carla Lisci gratefully acknowledges Fundação para a Ciência e Tecnologia (FCT) for the grant SFRH/BD/149699/2019 co-funded by the European Social Fund (ESF) and MEC national funds. Fabio Sitzia gratefully acknowledge the Recursos Humanos Altamente Qualificados for the contract with Ref. ALT2059-2019-24. The authors gratefully acknowledge the following funding sources: Fundação para a Ciência e Tecnologia (FCT) under the project UID/Multi/04449/2013 (POCI-01-0145-FEDER-007649), and Fundacao para a Ciencia e Tecnologia (FCT) under the project UIDB/04449/2020 and UIDP/04449/2020. The authors gratefully acknowledge the European Union - Portuguese Recovery and Resilience Plan, Grant Contract number: Sustainable Stone by Portugal, Call: 2021-C05i0101-02—agendas/alianças mobilizadoras para a reindustrialização - PRR, Proposal: C632482988-00467016.

## Ethics statement

Authors have conduct themselves with integrity, fidelity, and honesty. Authors will openly take responsibility for their actions and only make agreements which they intend to keep. Authors will not intentionally engage in or participate in any form of malicious harm to another person or animal.

## Data availability statement

The data that support the findings of this study are available from the corresponding author, Pires, V., upon reasonable request.

## CRediT authorship contribution statement

**Vera Pires:** Writing – review & editing, Writing – original draft, Validation, Supervision, Methodology, Investigation, Formal analysis, Data curation, Conceptualization. **Fabio Sitzia:** Writing – review & editing, Validation, Methodology, Investigation, Conceptualization. **Carla Lisci:** Writing – review & editing, Visualization, Validation, Methodology, Investigation, Formal analysis, Conceptualization. **Licinio Cordeiro:** Visualization, Validation, Resources.

## Declaration of competing interest

The authors declare that they have no known competing financial interests or personal relationships that could have appeared to influence the work reported in this paper.
